# Blastomycosis Complicated by Adult Respiratory Distress Syndrome in an Immunocompetent Adult: A Case Report and Literature Review

**DOI:** 10.7759/cureus.52319

**Published:** 2024-01-15

**Authors:** Alan M Furlan, Francisco F Costa Filho, Donald W Gusfa, Hansen M Tang, Benjamin S Avner

**Affiliations:** 1 Internal Medicine, Western Michigan University Homer Stryker M.D. School of Medicine, Kalamazoo, USA

**Keywords:** fungal lung infection, immunocompetent fungal, ards (adult respiratory distress syndrome), pulmonary blastomycosis, blastomycosis

## Abstract

Blastomycosis is an endemic mycotic infection caused by inhalation of thermally dimorphic fungi from the genus *Blastomyces*. *Blastomyces dermatitidis* is the species most related to human infection in the USA and North America. Adult respiratory distress syndrome (ARDS) is a rare complication of blastomycosis and is associated with high mortality. Due to its rarity, evidence-based guidelines for diagnosing and treating ARDS associated with blastomycosis are scarce.

In this case presentation, a 22-year-old male with a history of chronic cannabis use presented with severe respiratory symptoms, initially treated as community-acquired pneumonia. Despite antibiotic treatment, his condition deteriorated, necessitating intubation and resulting in the development of ARDS. A delayed diagnosis of pulmonary blastomycosis was confirmed through polymerase chain reaction testing. Treatment with amphotericin B and corticosteroids proved successful in addressing the fungal infection, leading to the recovery of the patient from his severe clinical condition. This case highlights the challenges associated with diagnosing and treating blastomycosis, particularly when complicated by ARDS, emphasizing the importance of considering fungal infections in the differential diagnosis of non-responsive pulmonary infections. Additionally, it suggests the potential utility of corticosteroids in severe cases and emphasizes the crucial role of early diagnosis and a combination of diagnostic modalities for the timely management of this rare and potentially life-threatening condition.

## Introduction

Blastomycosis, a mycotic infection caused by the thermally dimorphic fungus *Blastomyces*, is a significant concern in the eastern United States due to its endemic nature, resulting in a substantial number of cases and the potential for a high mortality rate, particularly in severe cases [1]. *Blastomyces dermatitidis* is the primary species responsible for a variety of human infections and manifestations, with a predominant pulmonary involvement but the capacity to affect multiple organs [2]. Although relatively rare, the emergence of adult respiratory distress syndrome (ARDS) presents a distressing and often fatal complication associated with this fungal infection.

There is a lack of documented data and evidence-based guidelines for managing ARDS in the context of blastomycosis-related ARDS, especially with regard to the use of corticosteroids. In light of this, we present a case of a 22-year-old immunocompetent male patient who developed ARDS due to pulmonary blastomycosis. The patient was initiated on amphotericin treatment after the failure of initial and subsequent treatments for community-acquired pneumonia, and he was later diagnosed with severe pulmonary blastomycosis during his hospitalization. A definitive diagnosis was achieved through polymerase chain reaction (PCR) analysis of bronchoalveolar lavage (BAL) fluid. The patient's clinical course was marked by a successful intervention, which included not only antifungal therapy but also the addition of systemic corticosteroids.

In this case report, our objective is to shed light on the diagnostic challenges in achieving a definitive diagnosis of blastomycosis, the treatment strategies employed, and our experience in successfully managing cases of ARDS associated with blastomycosis, including the use of corticosteroids. This case highlights the critical significance of timely clinical suspicion and effective management of this rare, yet life-threatening condition.

## Case presentation

The case involves a 22-year-old immunocompetent male who initially presented to the emergency department (ED) with a one-week history of shortness of breath and a persistent cough. One week prior to this visit, he had visited the same ED with pleuritic chest pain, productive cough, and shortness of breath. He was diagnosed with community-acquired pneumonia (CAP) and prescribed amoxicillin 500 mg every eight hours and doxycycline 100 mg every 12 hours for seven days. However, his symptoms worsened after starting antibiotics, leading to his return to the ED. The patient had a history of chronic cannabis use and reported a significant weight loss of 50 pounds and intermittent cough over the past two months. He denied any known contact with sick individuals. He worked two jobs, one as a college dormitory cleaner and the other constructing semi-permanent event sites, which involved rural farmland work. The onset of weight loss occurred soon after starting his construction job. There was no known family history of pulmonary diseases or immunodeficiency.

**Figure 1:**
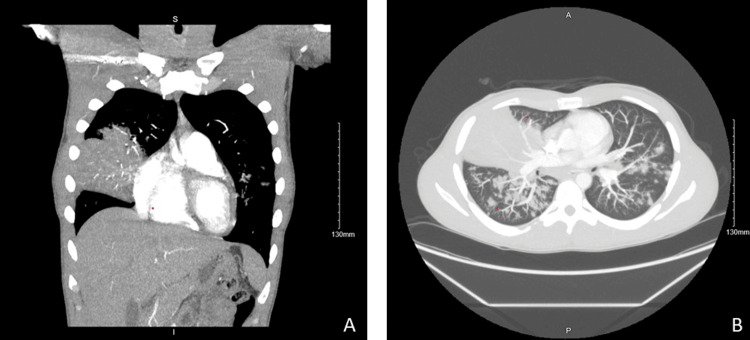
Computed tomography scan pulmonary embolism protocol There is extensive consolidation throughout the right middle lobe. Multiple air bronchograms are present. Correlate for symptoms of pneumonia. Some of this consolidation also extends into the inferior aspect of the right upper lobe. In addition, patchy ill-defined nodular airspace opacities are scattered throughout both lungs, the right greater than the left. Diffuse, atypical bronchopneumonia is suspected. A: coronal view; B: axial view.

Initial workup revealed a leukocytosis with a neutrophil predominance (17,000 WBCs per microliter), a positive urine drug test for cannabis, and a repeat chest X-ray showing consolidating right middle lobe pneumonia. He was admitted and started on IV ceftriaxone 1 g daily and azithromycin 500 mg daily. Further tests, such as a respiratory infectious disease panel, *Legionella* and *Streptococcus pneumoniae* antigen tests, serum beta-D glucan, HIV screen, sputum culture, and blood culture, all yielded negative results, except for *Mycoplasma pneumoniae* IgM antibody, considered equivocal after it was repeated (Tables 1, 2). A chest CT scan showed multifocal pneumonia with extensive consolidation in the right middle lobe (Figure 1). After three days of his initial therapy, the antibiotic regimen was broadened to vancomycin, dosed per pharmacy, and cefepime 2 g every eight hours due to clinical deterioration, including worsening respiratory status and persistent fever.

**Table 1:** Viral evaluation work-up PCR: polymerase chain reaction.

Test	Results
Adenovirus	Negative
Chlamydophila pneumoniae	Negative
Coronavirus 229E PCR	Negative
Coronavirus HKU1	Negative
Coronavirus NL63	Negative
Coronavirus OC43	Negative
Influenza A	Negative
Influenza B	Negative
Metapneumovirus	Negative
Parainfluenza 1	Negative
Parainfluenza 2	Negative
Parainfluenza 3	Negative
Parainfluenza 4	Negative
Respiratory syncytial virus	Negative
Rhinovirus/enterovirus	Negative
SARS-CoV-2	Negative

**Table 2:** Bacterial and mycobacterial culture work-up MTB: Mycobacterium tuberculosis; PCR: polymerase chain reaction; TB: tuberculosis; CMV: cytomegalovirus; MRSA: methicillin-resistant Staphylococcus aureus.

Test	Latest reference range & units	Results
MTB complex PCR	Not applicable	Negative
QuantiFERON-TB Gold Plus	Negative	Negative
TB1 antigen minus NIL	IU/mL	0.02 (negative)
TB2 antigen minus NIL	IU/mL	0.00 (negative)
Mitogen minus NIL	IU/mL	2.04 (negative)
NIL result	IU/mL	0.10 (negative)
Blood culture		Negative 3 times
Respiratory culture		Negative
MRSA screening (nares)		Negative
Streptococcus antigen		Negative
Mycoplasma pneumonia		Negative
Bordetella pertussis/parapertussis		Negative
Mycoplasma IgM		Positive/equivocal in the second test
Mycoplasma IgG		Negative

Additional workup for infectious diseases during the first week of hospitalization included tests for *Histoplasma* urine antigen and serum antibodies, *Blastomyces* serum antibodies, and repeat sputum culture. Before undergoing bronchoscopy with BAL, the patient received a dose of dexamethasone, which provided temporary relief from cough and fever (Table 3).

**Table 3:** Fungal evaluation work-up BAL: bronchoalveolar lavage; PCR: polymerase chain reaction.

Fungus tested/sample	Results
Histoplasma antigen result (BAL/blood antigen)	Negative
Fungitell quantitative (BAL)	<31 (negative)
Fungitell qualitative (BAL)	Negative
Pneumocystis (BAL)	Negative
Blastomyces dermatitidis (fungal culture PCR from BAL)	Positive 2x

**Figure 2:**
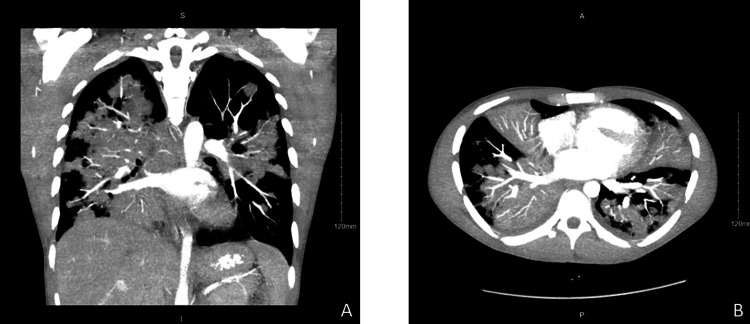
Computed tomography scan pulmonary embolism protocol Significant consolidation is noted in the bilateral lungs (right greater than left), which appears slightly worsened compared to the prior examination. A: coronal view; B: axial view.

*Blastomyces* antibody testing via enzyme immunoassay (EIA) returned a positive result on the seventh day of hospitalization. Liposomal amphotericin B 5 mg/kg daily was initiated for the invasive fungal infection, and vancomycin and cefepime were discontinued after three days of treatment. The patient's transbronchial biopsy revealed necrotizing granulomatous infiltrates, but Grocott methenamine silver staining did not detect fungal forms. As the patient's condition worsened, a new pulmonary CT scan was ordered, revealing radiological deterioration (Figure 2). On the 10th day of hospitalization, he was intubated, subsequently developing ARDS and experiencing radiological worsening. Although the *Blastomyces* confirmatory serology by immunodiffusion was negative, amphotericin B treatment was continued due to the severity of the patient's condition. Intravenous methylprednisolone 40 mg every eight hours was initiated. Subsequent testing, including a repeat bronchoscopy with BAL and PCR for *Blastomyces*, confirmed the fungal infection.

Following the initiation of methylprednisolone, the patient's clinical status improved, and he was extubated within 48 hours. The planned treatment included weaning off steroids and continuing liposomal amphotericin for 14 days before transitioning to oral itraconazole for at least six months of treatment. In our patient, amphotericin B was stopped due to intolerable side effects after 12 days of therapy, and itraconazole was started.

## Discussion

Like other systemic endemic mycoses, blastomycosis frequently poses a challenge for diagnosis and treatment for clinicians at the bedside. Prompt diagnosis of invasive fungal infections is essential to avoid delays in antifungal treatment and improve the patient's prognosis. ARDS is a rare complication of blastomycosis and is associated with high mortality rates. Our case describes a 22-year-old immunocompetent man with pulmonary blastomycosis complicated by ARDS during his hospitalization, and the definitive diagnosis was delayed until after a second BAL evaluation.

Currently, laboratory methods for diagnosing blastomycosis include fungal culture, histopathology, antigen detection, antibody tests, and nucleic acid amplification tests [3]. Fungal culture remains the "gold standard" for blastomycosis diagnosis. In cases of pulmonary blastomycosis, the yield of culture from invasive bronchoscopy is as high as 92% [4]. Specimens for culture can also be obtained from sputum, cerebrospinal fluid, or tissue biopsy samples. However, fungal colonies take an average of five to 14 days to develop, and when the infection burden is low, growth may take even longer [4]. In our case, BAL culture tested positive for *B. dermatitidis* after 18 days. Therefore, in severe cases with a high suspicion of fungal infection, prompt treatment should be initiated.

Non-culture-based laboratory diagnostic tests have been developed to overcome the time required for culture. Antigen detection using an EIA has proven to be useful for the rapid diagnosis of blastomycosis from samples such as urine, serum, BAL, or CSF [5]. This assay detects galactomannan present in the cell wall of *B. dermatitidis*. Previous literature has reported sensitivities ranging from 76% to 90% in urine and from 56% to 82% in serum [6,7].

Notably, in our case, the first laboratory result that raised suspicion of blastomycosis was a serologic test. Current guidelines, however, do not recommend using antibody testing (serology) such as complement fixation (CF) or immunodiffusion (ID) for blastomycosis diagnosis due to its poor sensitivity and specificity [7]. In our patient’s case, the serology did turn out to be beneficial in directing initial therapy while awaiting more specific test results. This illustrates the common challenges that clinicians face in such cases. Antifungal therapy with lipid amphotericin B was initiated primarily due to the absence of a more convincing differential diagnosis and the lack of improvement with empiric broad-spectrum antibiotics alone. On the 11th day of hospitalization, without a definitive blastomycosis diagnosis, a repeat BAL was performed, and *Blastomyces* PCR testing was ordered, yielding a positive result. The *Blastomyces* antigen testing and the BAL fungal cultures both also eventually resulted positive, further supporting the diagnosis that had been made by PCR.

It has been demonstrated that isolated cultures and PCR assays [8-10] achieved 100% specificity and sensitivity for *B. dermatitidis*, with 86% and 89% specificity and sensitivity, respectively, from clinical specimens [11]. This compares favorably to culture results. The first confirmatory blastomycosis diagnosis in our patient was a positive PCR from the BAL sample, which was obtained on the 15th day of hospitalization. This highlights the potential utility of a rapid, real-time PCR assay for the swift diagnosis of pulmonary blastomycosis.

The standard treatment for moderate or severe blastomycosis typically involves intravenous lipid amphotericin B for 10 to 14 days, followed by oral itraconazole [12]. While the Infectious Diseases Society of America (IDSA) guidelines do not address the use of corticosteroid therapy in this context, the American Thoracic Society (ATS) suggests considering corticosteroids for patients with severe hypoxemia. Severe pulmonary blastomycosis with an inflammatory reaction leading to ARDS is associated with high mortality rates [13,14]. Some reports suggest potential benefits of systemic corticosteroids in such cases [15-17] although robust evidence supporting this practice is lacking. Expert recommendations favor methylprednisolone at a dosage of 1 mg/kg/day for seven to 10 days for severely ill patients [18,19]. In the described case, the patient received a modified steroid regimen due to initial suspicions of autoimmune disease before confirming the blastomycosis diagnosis.

## Conclusions

This is a complex case involving a 22-year-old immunocompetent patient diagnosed with invasive pulmonary blastomycosis, complicated by ARDS, necessitating an invasive respiratory airway and mechanical ventilation. This case highlights the challenges in blastomycosis diagnosis and managing severe complications. Fungal infections present diagnostic difficulties due to nonspecific clinical symptoms and the necessity for specialized and invasive laboratory work-up. Despite initially being treated for CAP, suspicion of an invasive fungal infection prompted the initiation of IV liposomal amphotericin B treatment before confirmatory testing. A positive PCR test from a BAL eventually confirmed *Blastomyces* infection. The use of systemic corticosteroids, despite lacking evidence-based guidelines, demonstrated clinical improvement in this case and others in the literature, suggesting potential benefits in patient outcomes. This underscores the importance of considering fungal infections in non-responsive pulmonary infections, the challenges in diagnosis and treatment, and the ongoing debate surrounding corticosteroid use for severe fungal infections. Further research is essential to establish guidelines, emphasizing individualized evaluation before considering high-dose corticosteroids for severe pulmonary involvement in fungal infections.
